# Recent advances toward a molecular mechanism of efflux pump inhibition

**DOI:** 10.3389/fmicb.2015.00421

**Published:** 2015-05-05

**Authors:** Timothy J. Opperman, Son T. Nguyen

**Affiliations:** Microbiotix, Inc., Worcester, MAUSA

**Keywords:** efflux pump inhibitor, mechanism of action, RND family pumps, AcrB, AcrD, MexB, *Escherichia coli*, *Pseudomonas aeruginosa*

## Abstract

Multidrug resistance (MDR) in Gram-negative pathogens, such as the Enterobacteriaceae and *Pseudomonas aeruginosa*, poses a significant threat to our ability to effectively treat infections caused by these organisms. A major component in the development of the MDR phenotype in Gram-negative bacteria is overexpression of Resistance-Nodulation-Division (RND)-type efflux pumps, which actively pump antibacterial agents and biocides from the periplasm to the outside of the cell. Consequently, bacterial efflux pumps are an important target for developing novel antibacterial treatments. Potent efflux pump inhibitors (EPIs) could be used as adjunctive therapies that would increase the potency of existing antibiotics and decrease the emergence of MDR bacteria. Several potent inhibitors of RND-type efflux pump have been reported in the literature, and at least three of these EPI series were optimized in a pre-clinical development program. However, none of these compounds have been tested in the clinic. One of the major hurdles to the development of EPIs has been the lack of biochemical, computational, and structural methods that could be used to guide rational drug design. Here, we review recent reports that have advanced our understanding of the mechanism of action of several potent EPIs against RND-type pumps.

## Introduction

The rise of multidrug resistant (MDR) Gram-negative pathogens poses a significant clinical problem. Apart from the acquisition of acquired resistance traits, such as transposons and plasmids encoding proteins that inactivate antibiotics, many of these organisms have increased resistance resulting from mutations that alter the expression of genes that are involved in intrinsic resistance to antibiotics (Nikaido and Pages, 2012). One of the major contributors to intrinsic resistance in Gram-negative bacteria are efflux pumps of the Resistance-Nodulation-Division (RND) family efflux in Gram-negative bacteria, which extrude a broad spectrum of antibiotics and biocides, including the fluoroquinolones (e.g., ciprofloxacin and levofloxacin), β-lactams (e.g., piperacillin, meropenem, and aztreonam; Piddock, 2006), tetracyclines (minocyline), oxizolidinines (linezolid), and β-lactamase inhibitors (e.g., clavulanate and sulbactam; Li et al., 1998; Nakae et al., 2000), from the periplasm to the outside of the cell. Because of their broad-substrate specificity, overexpression of the RND efflux pumps results in decreased susceptibility to diverse array of antibacterial agents and biocides (Nikaido and Pages, 2012). In addition, elimination of RND pumps in *Pseudomonas aeruginosa* by genetic deletion (Lomovskaya et al., 1999) or inhibition with a potent efflux pump inhibitor (EPI; Lomovskaya et al., 2001) decreases the frequency of resistance to levofloxacin. In *Escherichia coli*, a functional RND pump (AcrAB-TolC) is required for the selection of mutations in the targets of fluoroquinolones (*gyrA* and *gyrB*) that give rise to fluoroquinolone resistance (Singh et al., 2012). Furthermore, RND pumps have been shown to play a role in virulence of the enteric pathogen *Salmonella enterica* serovar Typhimuirum (Nishino et al., 2006), and EPIs that target RND pumps have been shown to inhibit biofilm formation in *E. coli* and *Klebsiella pneumoniae* (Kvist et al., 2008). Therefore, EPIs could be useful as adjunctive therapies with an antibiotic to improve antibacterial potency at low antibiotic concentrations, reduce the emergence of resistance, inhibit biofilm formation, and decrease virulence of enteric pathogens. Consequently, there has been considerable interest in developing EPIs of the RND family pumps.

Several potent EPIs that target the RND family pumps have been described in the literature (Van Bambeke et al., 2006), however, none have reached clinical development. A family of peptidomimetics, including PAβN (MC-207 110), that exhibited potent inhibition of efflux pumps in *P. aeruginosa* has been developed for use as an adjunctive therapy (Renau et al., 1999, 2001, 2002, 2003; Lomovskaya et al., 2001; Watkins et al., 2003). Some of these inhibitors were validated using *in vivo* infection models (Renau et al., 1999, 2001; Watkins et al., 2003), however, they were abandoned because of toxicity (Lomovskaya and Bostian, 2006). The positive charged moieties that were required for activity in *P. aeruginosa* caused nephrotoxicity. In addition, a series of pyridopyrimidine EPIs that are specific for the MexAB efflux pump of *P. aeruginosa* was advanced to the preclinical stage (Nakayama et al., 2003a,b, 2004a,b; Yoshida et al., 2006a,b, 2007). However, the development of this series appears to have been halted. Finally, a pyranopyridine EPI, MBX2319, with potent activity against Enterobacteriaceae, but low activity vs. *P. aeruginosa*, has been described recently (Opperman et al., 2014). This compound is in the early stages of lead optimization and has not been tested for efficacy in animal models of infection. The difficulties encountered during the development of these varied classes of EPIs underscore some of the most significant challenges for developing EPIs for clinical use: potency, spectrum of activity, pharmacokinetics, and toxicity. Detailed knowledge of the mode of inhibition and the binding site of an EPI could facilitate the rational design of analogs with properties that could address these challenges.

There are significant challenges to mechanism of action (MOA) studies for EPIs vs. the RND family efflux pumps, many of which complicate their discovery and development. One of the major challenges is the structural complexity of the RND pumps, which are an integral membrane (IM) molecular machine that spans the inner membrane, periplasmic space, and the outer membrane. The RND family efflux pumps comprise a tripartite structure, consisting of an IM efflux transporter with broad-substrate specificity, an outer membrane channel that carries substrates from the pump through the outer membrane, and a periplasmic adapter protein. The major RND pumps in *E. coli* and *P. aeruginosa* are AcrAB-TolC and MexAB-OprM, respectively; AcrB/MexB are the IM pumps, AcrA/MexA are the membrane adapter protein, and TolC/OprM form the channel through the outer membrane. The pump subunits (AcrB and MexB) consist of the following domains: (1) an IM domain comprised of 12 transmembrane helices that utilizes proton-motive force to drive the pumping action; (2), a porter domain comprised of the two large periplasmic loops that binds and extrudes substrates, and (3) a cap domain that binds to TolC [reviewed in Blair and Piddock (2009), Eicher et al. (2009), Pos (2009), Nikaido and Pages (2012)]. The three-dimensional structure of AcrB was first described as a symmetrical trimer, in which all subunits were in the same conformation (Murakami et al., 2002). Subsequently, an asymmetrical three-dimensional structure of AcrB was described (Murakami et al., 2006; Seeger et al., 2006; Sennhauser et al., 2007), in which the conformation of each subunit was different. Several lines of evidence indicate that the asymmetrical structure represents the biologically relevant form of the pump (Seeger et al., 2008; Takatsuka and Nikaido, 2009; Eicher et al., 2014). The conformations of the three subunits of the pump have been described as the Access (loose), Binding (tight), and Extrusion (open) subunits. The current model for the mechanism of the RND pumps is that each conformation represents a distinct step in the translocation pathway, in which each subunit successively assumes each of the conformations as substrates first interact with the pump in the Access conformation, are moved to the substrate binding pocket in the Binding conformation, and are then extruded into a central channel that leads to TolC as the binding pocket collapses en route to the Extrusion conformation. The entire process is driven by proton-motive force that is transduced by the IM domain of the protein (Su et al., 2006; Seeger et al., 2009; Takatsuka and Nikaido, 2009).

Of particular interest to the design of EPIs is the structure of the substrate binding pocket in the Binding protomer, also known as the distal binding site. The broad-substrate specificity of AcrB and MexB suggested that the substrate binding site would exhibit unique features that enable polyspecific, but not non-specific, binding of substrates. The substrate binding pocket was clearly defined when the three-dimensional structures of the asymmetric AcrB trimer with two pump substrates, minocycline and doxorubicin, bound to the Binding protomer were reported (Murakami et al., 2006). The substrate binding pocket comprised a large cavity lined with hydrophobic (Phe136 and Phe178, Phe610, Phe615, Phe617, and Phe628) and polar residues (Asn274 and Gln176). Minocycline and doxorubicin interacted with a distinct set of amino acid residues that line the deep binding pocket in the “Binding” subunit. Both compounds interacted mainly with hydrophobic residues through Van der Waals and ring-stacking interactions, and made hydrogen bonding interactions with the polar residues. However, minocycline and doxorubicin bound to slightly different regions of the binding pocket. Thus, the structure of the binding pocket with substrates explains the broad-substrate specificity of the pump, which is characterized by interactions based on generalized physical properties, such as hydrophobicity, and low binding affinities. Consequently, developing inhibitors that can bind to this site with high affinity is a major challenge.

Despite complexities of the RND family pumps, progress toward a better understanding of the MOA of several EPIs has been made on several fronts. In this article, we will review two aspects of the MOA studies of the major EPIs. Thus, the review will be divided in two parts. First, we will review the various technologies that have been used to study the structure and function of RND family efflux pumps and how they have been, or could be applied to study the mechanism of EPIs. The technologies that will be discussed include genetic, biochemical, structural, and computational approaches. Second, we will review the major classes of EPIs in terms of their biological activities, stage of development, and what is known about their MOA, including at least three important developments. These include the first crystal structure of an EPI bound to two RND pumps (Nakashima et al., 2013), the first report of EPI-resistant mutants that map to the binding site of AcrB (Schuster et al., 2014), and the application of molecular dynamic (MD) simulations to model the binding sites of other EPIs (Vargiu and Nikaido, 2012; Vargiu et al., 2014).

## Approaches to Study the Mechanism of Action of EPIs

### Genetic Studies

The classic method for identifying the molecular target of an antibacterial agent is the select for resistant mutants and to map the mutations to the putative target. However, in the case of EPIs, this approach has proven to be very difficult for the following reasons. Because the majority of EPIs do not exhibit antibacterial activity, resistant mutants cannot be selected directly. Instead, mutants resistant to the EPI must be selected in the presence of an antibiotic or biocide that is a substrate of the pump, which provides the selective pressure. The selection for EPI-resistant mutants usually consists of an EPI and antibiotic at concentrations that would enable growth if a mutation resulted in resistance to the EPI. This selection scheme creates two problems. First, since the EPI and the antibiotic bind to the substrate binding pocket, it is possible that mutations result in decreased affinity for the EPI also reduce affinity for the antibiotic. This is particularly problematic for EPIs that are pump substrates or competitive inhibitors that bind to the same or an overlapping site in the binding pocket. Second, the presence of an antibiotic in the selection results in a high background of mutants with decreased susceptibility to the antibiotic due to mutations in genes that encode the antibiotic target or another intrinsic resistance mechanism, such as antibiotic influx (porin). Third, if EPI-resistant mutants are obtained in such a screen, they may be specific for the antibiotic used for selection. Finally, it is likely that the structure of the substrate binding pocket of the RND family pumps, which is large and contains potentially overlapping binding sites, decreases the probability of identifying resistant mutants. Indeed, site-directed mutations affecting the residues that were shown to interact with minocycline or doxorubicin in co-crystal structures do not significantly affect the minimal inhibitory concentrations (MICs) for these drugs, suggesting a plasticity in their interactions with the substrate binding pocket (Bohnert et al., 2008). Consequently, there have been no reports of the isolation of spontaneous EPI-resistant mutations in RND-family pumps (Lomovskaya and Bostian, 2006).

Site-directed mutagenesis and construction of hybrid pumps were used to map the substrate binding domain and the determinants of binding specificity. To map the substrate binding sites and determinants of substrate specificity, Elkins and Nikaido (2002) constructed chimeric pumps in which the periplasmic loops of AcrD and AcrB were replaced with the corresponding loops of AcrB and AcrD, respectively. The resulting chimeras exhibited the substrate specificities characteristic of the pump that was the source of the periplasmic loops, indicating that the functions of substrate binding and specificity reside in the periplasmic loops. The exchange of periplasmic loops between the following AcrB homologs yielded similar results: *P. aeruginosa* MexB and MexY (Eda et al., 2003), and AcrB and MexB (Tikhonova et al., 2002). Mao et al. (2002) selected mutants of AcrD with expanded substrate recognition, which mapped to the periplasmic loops comprising the porter domain. Kobayashi et al. (2014) replaced portions of the access binding site of AcrB with corresponding non-conserved portions of AcrD to identify residues that are responsible for the selectivity of AcrD for anionic β-lactam antibiotics, such as aztreonam, carbenicillin, and sulbenicillin. Building on the results of these studies, the authors used site-directed mutagenesis to construct an AcrB pump that conferred high-level resistance to anionic β-lactam antibiotics, a characteristic of AcrD. In addition, site-directed mutants of AcrB have been constructed to map residues in the deep binding pocket that play a key role in substrate recognition and extrusion (Bohnert et al., 2008). However, these types of experiments have not been used to determine residues important for the binding of EPIs.

In contrast, mutations responsible for EPI resistance were identified in *bmr*, a broad-substrate specificity efflux pump in *Bacillus subtilis* of the major facilitator superfamily (MFS), that reduced affinity for an inhibitor (reserpine), but did not affect affinity for the substrate (ethidium bromide; Ahmed et al., 1993). This was accomplished by randomly mutagenizing a strain carrying a plasmid-encoded copy of *bmr*, followed by a selection for mutants consisting of a serial passage in the presence of ethidium bromide and increasing concentrations of reserpine. The mutations were mapped to *bmr* and resulted in substitution of Val296 with Leu. The mutation reduced the affinity of reserpine for Bmr, but did not affect the efflux of ethidium bromide. It is not clear whether this mutation directly affects binding site or causes change in conformation that affects binding at a distance. Since the three-dimensional structure of *bmr* is not known, and the substrate binding site has not been identified, it is difficult to determine the significance of the resistance mutation. Nevertheless, this study demonstrated that it is possible to generate and select mutants in an efflux pump gene with reduced susceptibility to an EPI.

**FIGURE 1:**
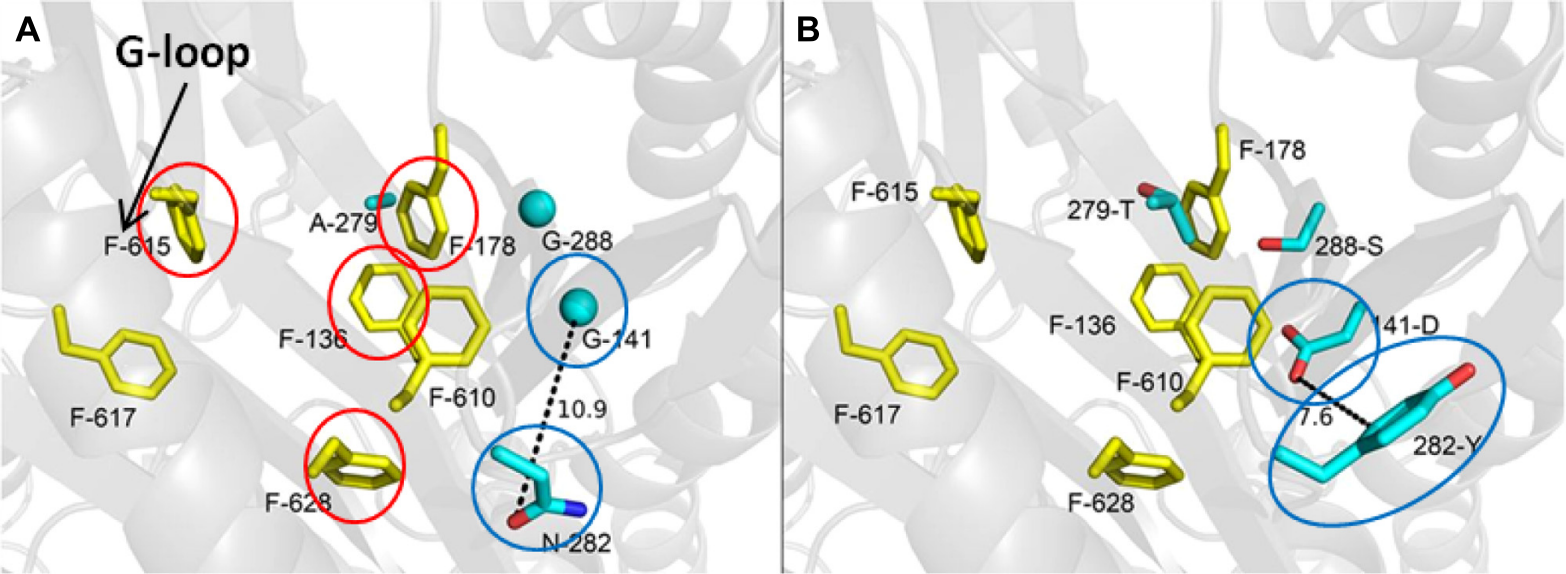
**The most frequently selected mutations that were isolated after *in vitro* random mutagenesis of the periplasmic domain of AcrB and linezolid/NMP selection are located in the distal binding pocket. (A)** The wild type distal binding pocket of AcrB. The side chains of the wild-type residues that are substituted in the NMP-resistant mutant are shown as cyan sticks (glycines as cyan spheres), and distal binding pocket phenylalanine side chains are shown as yellow sticks. The view of the distal binding pocket is a side view from the periplasmic outer face. The residues indicated with red circles are predicted to interact with NMP my molecular dynamic simulations (Vargiu et al., 2014). The location of the G-loop is indicated by the arrow. The residues indicated by the blue circles are those substituted in the G141D N282Y double mutant, which exhibits the strongest NMP-resistant phenotype. **(B)** The NMP-resistant distal binding pocket of AcrB. The residues indicated by the blue circles are those of the G141D N282Y double mutant. Reprinted with permission from Schuster et al. (2014).

The isolation of the first EPI-resistance mutations in an RND-family pump was reported more than 20 years after the *bmr* resistance mutations were reported. After attempts to isolate mutants in *E. coli* that were resistant to 1-(1-naphthylmethyl)-piperazine (NMP) and PAβN in a serial passage experiment, Schuster et al. (2014) randomly mutagenized the two large periplasmic loops of AcrB using error-prone PCR. They selected for mutants resistant to NMP and PAβN in combination with linezolid or clarithromycin, respectively, in a single step selection on agar plates. The authors isolated several mutants with reduced susceptibility to NMP that did not decrease the efflux activity of AcrB. When the NMP-resistant mutations were reconstructed using site-directed mutagenesis (alone and together), only a double mutant, in which Gly141 was substituted with Asp (G141D) and Asn282 was substituted with Tyr (N282Y), significantly affected susceptibility to NMP. As shown in **Figures 1A,B**, the mutated residues are located near the outer face of the distal substrate binding pocket near Phe610, which plays an key role in the extrusion process (Bohnert et al., 2008), and is across from the so-called “switch-loop” that separates the proximal (access) and the distal (substrate) binding sites (Nakashima et al., 2011; Eicher et al., 2012). The authors speculated that the G141D N282Y amino acid substitutions prevent the binding of NMP to the distal binding pocket. However, the substituted residues are not near the binding site of NMP that has been predicted by computational methods (Vargiu et al., 2014; see **Figure 1A**, and below for discussion), suggesting that the mutated residues may interfere with the action of NMP through an alternative mechanism. Interestingly, the mutated residues reduce susceptibility to NMP in the presence of linezolid only, the antibiotic used for selection. This strongly suggests that EPI-resistant mutants isolated in this type of screen are specific to the antibiotic used for the selection, and that the location of the substituted residues cannot be directly interpreted as being part of the EPI binding site. Because it is not known whether the NMP-resistant mutant of AcrB has decreased affinity for NMP, it is possible that the G141D N282Y amino acid substitutions enable the extrusion of linezolid while NMP is bound to the deep binding pocket. Further experiments are needed to fully understand the significance of this EPI-resistant mutant.

Consistent with the experience of other groups, Schuster et al. (2014) were unable to isolate mutants with reduced susceptibility to PAβN, a substrate for RND-family pumps (Lomovskaya et al., 2001; Bohnert et al., 2008). For EPIs that are substrates or competitive inhibitors, mutations that alter the binding site of the EPI may also affect the affinity for substrates, which could result in cell death if an antibiotic is used for selection. In addition, the plasticity of the binding site (see above) may also affect EPIs that are substrates. Although there are only two examples, it is tempting to speculate that there is a correlation between the mode of inhibition of an EPI and whether it is possible to isolate EPI-resistant mutations that map to the RND pump.

### Biochemical Studies

As mentioned above, the RND pumps are complex IM machines, which complicate the biochemical analysis of these proteins. Nevertheless, the development and use of biochemical systems for the study of the RND pumps have been reported, however, none have been used to analyze the mode of inhibition of EPIs. These studies are summarized below.

#### Enzyme Activity Assays

Zgurskaya and Nikaido (1999) reported a biochemical system in which purified AcrB was reconstituted into proteoliposomes. Fluorescent derivatives of the lipids phosphatidylethanolamine labeled with NBD or rhodamine, which are AcrB substrates, were incorporated into proteoliposomes at concentrations high enough to for NBD fluorescence to be quenched by rhodamine. To prevent reincorporation of the substrate into proteoliposome, unlabeled liposomes were added the reaction mixture to act as lipid traps for labeled lipid substrate. Therefore, efflux activity results in an increase in the fluorescence of NBD. The efflux activity was dependent on the presence of a transmembrane proton gradient, and was stimulated by addition of AcrA through an unknown mechanism. The addition of known pump substrates inhibited efflux of labeled lipids through competition. The bile salts taurocholate and glycocholate were the strongest inhibitors, with IC_50_ values ranging between 15 and 20 μM. While this biochemical system is amenable for the study of EPIs, it does not appear that this system has been used for this purpose. The reasons for this are not known. However, it is possible that there has been little interest in such assays, or that the assay is too difficult to implement.

#### Cell-Based efflux Pump Activity Assay

Nagano and Nikaido (2009) developed a cell-based assay that measures the efflux kinetics of the cephalosporin, nitrocefin, by the intact AcrAB-TolC pump. In this assay, the rate of nitrocefin hydrolysis by a periplasmic β-lactamase was measured spectrophotometrically. Using the Michaelis–Menten constants for the β-lactamase (K_M_ and V_max_), the rate of nitrocefin hydrolysis (V_h_) was used to determine the periplasmic concentration (C_p_) of nitrocefin. The rate of nitrocefin influx (V_in_) across the OM was determined experimentally as a function of the external concentration (C_o_). The difference between *V*_in_ and the measured rate of cephalosporin hydrolysis (V_h_) corresponds to the rate of efflux (V_e_) by AcrAB-TolC. By plotting V_e_ against C_p_ for nitrocefin, a curve that is indicative of Michaelis–Menten type kinetics was obtained, from which a K_M_ (6 μM) and Vmax (0.024 nmol/mg/s) were calculated. This assay format is broadly applicable to any β-lactam antibiotic that is a substrate of the periplasmic β-lactamases of *E. coli* (AmpC or TEM-1). Lim and Nikaido (2010) used this assay to determine the kinetic parameters for several penicillins and cephalosporins. Interestingly, the penicillins exhibited a stronger affinity for AcrAB-TolC than did the cephalosporins, and showed a kinetic behavior that indicated a strong positive cooperativity. Clearly, this assay format could have a wide utility in the analyses of efflux pump substrates and inhibitors.

The nitrocefin assay has been used to estimate the effects of an EPI (MBX2319) on the kinetic behavior of AcrAB-TolC (Opperman et al., 2014). The data demonstrated that MBX2319 (0.2 μM) inhibited AcrAB-TolC, and the inhibition was due mainly to a large (4.4-fold) increase in the K_M_. Higher concentrations of MBX2319 (1–10 μM) completely inhibited nitrocefin efflux and prevented kinetic analyses. In contrast, PAβN (1 μM) had no effect on the efflux of nitrocefin, suggesting that this compound does not directly affect AcrB function at lower concentrations. The data suggest that MBX2319 competes with nitrocefin for a site in the binding pocket, or decreases its access to the binding site. This assay has proven to be useful for determining the effects of EPIs on the kinetic parameters of AcrAB-TolC, and could possibly used to determine the mode of inhibition (competitive, non-competitive, and uncompetitive) of an EPI. In addition, the assay format could be adopted to measure the half maximal inhibitory concentration (IC_50_) of EPIs, which could be used to evaluate and prioritize experimental EPIs. The assay format has several advantages. It is versatile and does not require expensive reagents or equipment. However, because it is a cell-based assay, great care must be taken to control as many variables as possible to minimize variability. In addition, the calculations for V_in_ depend on several values (see Nagano and Nikaido, 2009) that may change depending on growth conditions or the bacterial strain that is used. Therefore, it is important to verify these values under the conditions that exist in your laboratory.

#### Binding Assays

Several research groups have reported binding assays designed to measure the affinity of various substrates to the purified AcrB or MexB. While many of these methods could be used to study the interactions between EPIs and RND family pumps, there are only a few reports of these types of experiments. In addition, great care must be taken when performing or interpreting the results of these experiments, as the RND pumps are large and have multiple hydrophobic sites where non-specific interactions could occur. Therefore, proper negative controls should be included in each experiment.

The most promising technology for binding studies involving AcrB is isothermal titration calorimetry (ITC). Nakashima et al. (2013) measured the binding parameters of D13-9001, a pyranopyrimidine EPI (see below), to purified AcrB, MexB, and MexY using ITC. The data for the titration curves of D13-9001 for AcrB and MexB showed saturation at a stoichiometry of 1:1 (compound: trimer), indicating specific binding interactions. The K_D_ values of D13-9001 binding AcrB and MexB were 1.15 and 3.57 μM, respectively. In contrast, no saturation binding in the titration curves for MexY, which is not inhibited by D13-9001, was observed. Another group used fluorescence polarization (FP) to study binding of substrate compounds, such as rhodamine 6G (5.5 ± 0.9 μM), ethidium bromide (8.7 ± 1.9 μM), ciprofloxacin (74.1 ± 2.6 μM), and proflavin (14.5 ± 1.1 μM), to AcrB (Su and Yu, 2007). However, neither of these studies included a negative control, such as a proton transduction deficient mutant, in which none of the subunits are in the Binding conformation. These results indicate that ITC and FP are useful tools for analyzing the affinity between EPIs and efflux pumps; however, they do not indicate where the EPIs bind to the pump.

An experimental approach has been described, however, that could be used to identify binding sites for EPIs. This approach utilizes a pump substrate, usually a fluorescent dye molecule that carries a moiety that acts as a cross-linking agent by forming a covalent bond with a specific amino acid side chain, such as the free sulfhydryl groups in Cys. This method was initially used to study the substrate binding specificity of the RND pump MexD by Mao et al. (2002). They used 2-(4′-maleimidylanilino)- naphthalene-6-sulphonic acid (MIANS) as the cross-linking reagent, which becomes fluorescent only after it reacts with sulfhydryl groups, which enables the cross-linking reaction to be monitored by increased fluorescence. Amino acid residues that were predicted to play a role in substrate recognition were substituted with Cys, and the effect of MIANS on efflux of various substrates was measured. The pump was engineered to contain a single Cys residue. This method was later used to map the entire substrate path in the AcrB pump of *E. coli* (Husain and Nikaido, 2010; Husain et al., 2011). In these studies, residues that were predicted to line the substrate path were substituted with a Cys residue using site-directed mutagenesis, and the accessibility of each of these residues to a lipophilic fluorescent cross-linking reagent (Bodipy-FL-maleimide) was measured. In principle, this assay system could be used to map the binding sites of EPIs through the competitive exclusion of a fluorescent-cross linking agent by the inhibitor upon binding to the same site (competitive), or to an allosteric site binding (either non-competitive or uncompetitive), which can be monitored by the accessibility of the Cys to the cross-linking reagent. The different modes of binding can be distinguished upon analysis of the binding kinetics. Indeed, this approach was used to obtain experimental support for hypotheses on the mechanism of substrate recognition in the deep binding pocket of AcrB, which were generated by *in silico* docking experiments (Takatsuka et al., 2010). Competition experiments were used to demonstrate that compounds predicted to bind to the “groove” of the substrate binding pocket compete with cross-linking reagent for a Cys residue in the groove, whereas, compounds predicted to bind lower in the site, or the so-called “cave,” did not compete. A covalently linked AcrB trimer, which would allow construction of mutant proteins in which a single subunit can assume the “binding” conformation, could prove to be useful in this type of study (Takatsuka and Nikaido, 2009; Zgurskaya, 2009). Despite the fact that this approach could be applied readily to the study of the interaction of EPIs with AcrB, it does not appear to have been used for this purpose. One problem with this approach is that it requires several AcrB variants, in which a single Cys substitution is introduced at several sites in the access and deep binding pockets of AcrB. In addition, the fluorescent cross-linking reagent must be a pump substrate. Fortunately, several such cross-linking compounds are available, such as MTS-rhodamine (Loo and Clarke, 2002), BODIPY-FL-maleimide (Husain et al., 2011), *N*-ethylmaleimide (Loo and Clarke, 2002; Guan and Kaback, 2007), or monobromobimane (Fluman et al., 2009). Thus, this approach could prove to be very useful for MOA studies for EPIs.

### X-ray Crystallography

Very few co-crystal structures of the asymmetric trimer of AcrB bound to substrates, and until recently, no co-crystal structures of an EPI bound to AcrB (see below) have been reported. As mentioned above, co-crystal structures for the substrates minocycline and doxorubicin bound to the deep binding pocket (Murakami et al., 2006) and erythromycin and rifampicin bound to the access binding site (Nakashima et al., 2011) of the asymmetric trimer have been reported. In contrast, several co-crystal structures of the symmetrical form of AcrB with substrates (ethidium bromide, rhodamine 6G, ciprofloxacin, nafcillin) and one EPI (PAβN; Yu et al., 2005), as well as linezolid (Hung et al., 2013), have been published. However, the relevance of these structures is difficult to interpret as the pump is not in the conformation are that is relevant biochemically. The paucity of co-crystal structures is probably the direct result of the difficulty in producing co-crystals. It is possible that the difficulties arise from the relatively low binding affinities of the substrates for the binding site.

**FIGURE 2:**
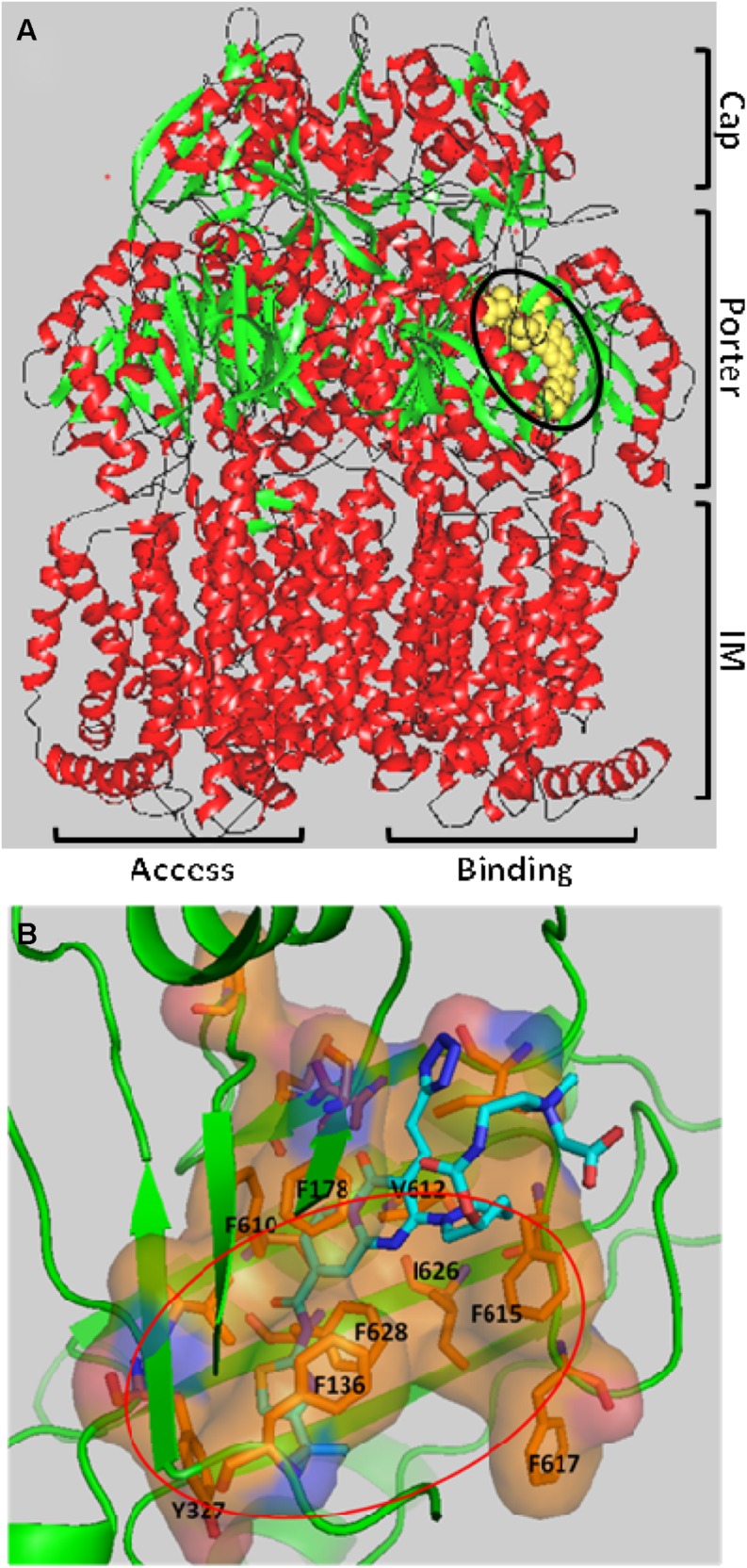
**The three-dimensional structure of the EPI D13-9001 bound to the Binding protomer of AcrB. (A)** D13-9001 bound to the Binding protomer of the asymmetric trimeric AcrB. D13-9001 is drawn using a yellow space model and is highlighted by the black oval. The integral membrane (IM), Porter, and Cap domains are indicated on the right side of the figure. The Binding and Access protomers are indicated at the bottom of the figure. The Extrusion protomer is not visible. Secondary structure is indicated as follows: alpha helix (red); beta sheet (green); Loops (black). The figure was generated by MarvinSpace (ChemAxon Ltd) using PDB ID: 3w9h (Nakashima et al., 2013). **(B)** A detailed view of the D13-9001 binding site. The location of hydrophobic trap is indicated by the orange oval, and amino side chains of residues that comprise the hydrophobic trap are shown. The molecular surface of the binding site has been colored based on hydrophobicity (orange) and hydrophilicity (blue). The D13-9001 structure is depicted as a stick drawing that is colored according to the atom type as follows: red, oxygen; yellow, sulfur; dark blue, nitrogen; cyan, carbon; white, hydrogen. This figure was made by Hiroshi Nikaido (UC Berkeley) using PyMol (Schrödinger).

Nakashima et al. (2013) reported a major breakthrough in the field when they published the first co-crystal structure of the pyridopyrimidine EPI D13-9001 (see **Figures 2A,B**) bound to AcrB (*E. coli*) and MexB (*P. aeruginosa*). The inhibitor, D13-9001, is a pyridopyrimidine compound that is a potent inhibitor of AcrB and MexB, but not of MexY (*P. aeruginosa*). The hydrophobic *tert*-butyl thiazolyl aminocarboxyl pyridopyrimidine moiety of D13-9001 binds tightly to a narrow depression, referred to as the hydrophobic trap, in the deep substrate binding pocket in the “Binding” protomer (see **Figures 2A,B**). The hydrophilic portion of the compound extends into to substrate binding groove where it interacts with polar residues. A detailed description of the binding site can be found below. The location of the binding site and the high affinity of D13-9001 suggested a MOA for this compound. The current hypothesis is that D13-9001 binds tightly to the hydrophobic trap and prevents the conformational changes that are needed for the proper activity of the pump (Nakashima et al., 2013). In addition, the hydrophilic portion of D13-9001 is expected to prevent binding of a wide range of substrates to the binding cleft. It is not clear why the co-crystal structure of D13-9001 is the only one that has been published. While it is not known how many other EPIs have been attempted, it is possible that D13-9001 has some properties that are amenable to X-ray crystallography, as relatively standard conditions for formation of co-crystals were used (Nakashima et al., 2013). However, the real significance of this work lies in the discovery of a binding site on AcrB for non-competitive (substrate) inhibitors that can be exploited for rational design of EPIs.

### Computational Methods

Because of the biochemical complexity of the RND family pumps and the difficulty of producing co-crystals, many researchers have turned to computational methods to explore the structure function relationships of AcrB and MexB. The computational systems that have been used to study RND family efflux pumps consist of algorithms that are designed to simulate the molecular interactions and conformational changes that comprise the current model for substrate recognition and extrusion. These systems range from relatively simple algorithms for docking compounds into the substrate binding pocket to complex MD simulations. Docking algorithms, such as AutoDock Vina (Trott and Olson, 2010), utilize the static binding site defined by a three-dimensional crystal or NMR structure in the absence of solvent to identify the most energetically favorable binding pose of a small molecule. In contrast, MD simulations attempt to reproduce the behavior of real molecules in motion in the presence of solvent. MD simulation consists of the numerical, step-by-step, solution of the classical equations of motion for each atom. A computer model of the protein is prepared from a three-dimensional protein structure, and the forces acting on each atom in the system atoms are then estimated using an equation that includes molecular ‘force fields,’ which model various bonded and non-bonded inter-atomic interactions. Several excellent reviews of the methodology of MD simulations for proteins have been published (Karplus and McCammon, 2002; Durrant and McCammon, 2011). Advances in technology have increased the predictive accuracy of these methods. For example, MD simulations of the enzyme-inhibitor complex between trypsin and the inhibitor benzamidine have produced binding predictions in which the root mean square deviation (rmsd) for the bound inhibitor was less than 2 Å as compared to the crystal structure (Buch et al., 2011). However, the current MD simulation methodologies are limited by high computational costs and by the approximations of the force fields used in the modeling.

The use of computational methods for the study of RND-family pumps and EPIs is made possible by the existence of high-resolution three-dimensional structures of RND-family pumps with and without substrates, such as AcrB (Murakami et al., 2006; Sennhauser et al., 2007; Nakashima et al., 2011, 2013) and MexB (Sennhauser et al., 2009; Nakashima et al., 2013), and supporting biochemical data. Takatsuka et al. (2010) used docking experiments to examine the interaction of 30 compounds, including minocycline, doxorubicin, tetracycline, and levofloxacin with the distal substrate binding site of the binding protomer. They found that the substrates that were tested are predicted to bind to one of two regions of the binding pocket: a narrow groove at the distal end of the pocket, and a wider proximal region, which they called the cave region. The results of biochemical studies, including competition with the substrate nitrocefin and the fluorescent cross-linking dye fluorescein-5-maleimide (see above), provided support for the predictions of the docking experiment. Several studies that employ MD simulations to study the mechanism of substrate recognition and extrusion in AcrB and MexB have been reported. For example, MD simulations have been used to examine the mechanism of the peristaltic action of AcrB (Schulz et al., 2010; Yao et al., 2010; Fischer and Kandt, 2013; Schulz et al., 2015), to map the path of substrate from the periplasm to the central cavity (Imai et al., 2011), to study the role of water in substrate extrusion (Schulz et al., 2011) and proton transfer in the transmembrane domain (Fischer and Kandt, 2011), and the role of a key residue Phe 610 in extrusion (Vargiu et al., 2011). Several of these studies have been reviewed previously (Ruggerone et al., 2013a,b; Fischer et al., 2014). Because of the limitations posed by docking, MD simulations have been used to study substrate binding and specificity of AcrB (Vargiu and Nikaido, 2012; Yao et al., 2013) and MexB (Collu et al., 2012). In particular, Vargiu and Nikaido (2012) used MD simulations to examine the interaction between nine substrates and the distal binding pocket of AcrB in terms of the binding energy, hydrophobic surface-matching, and the residues involved in the process. Despite the potential inaccuracies resulting from the limitations of the system, the MD simulations enabled the assessment of the contribution of various residues in ligand binding and produced a much more realistic picture of the interaction of various ligands with AcrB, as compared to docking. These limitations include the following: (1) an inability to model the effect of the local environment in the binding site on the charge states of substrates, (2) the difficulties in estimating binding affinities due to the intrinsic limitations of the methodology used to calculate force fields, and (3) the absence of accessory proteins, such as AcrA (Nikaido, 2011) and AcrZ (Hobbs et al., 2012; Du et al., 2014), which may be required to form the active conformation of AcrB. The major advantages of the MD simulations include the presence of water molecules in the simulation and the flexibility of the distal binding site, which enables the modeling of binding interactions that are more realistic. For example, the binding site predicted for the bile salt taurocholate by MD simulations was similar to the one predicted by docking. However, MD simulation oriented the compound so that the hydrophobic and hydrophilic groups on the different sides of the planar structure were facing hydrophobic binding site residues or the solvent filled channel, respectively. Strong interactions between water molecules and most of the other substrates were observed, which are indicative of a more realistic binding prediction. This is illustrated by the binding pose of minocycline that was predicted by the MD simulation, which was in very good agreement with the crystallographic structure (Murakami et al., 2006; Eicher et al., 2012). The results of these MD simulations demonstrate that this is a promising approach for studying substrate binding to RND-family pumps.

**FIGURE 3:**
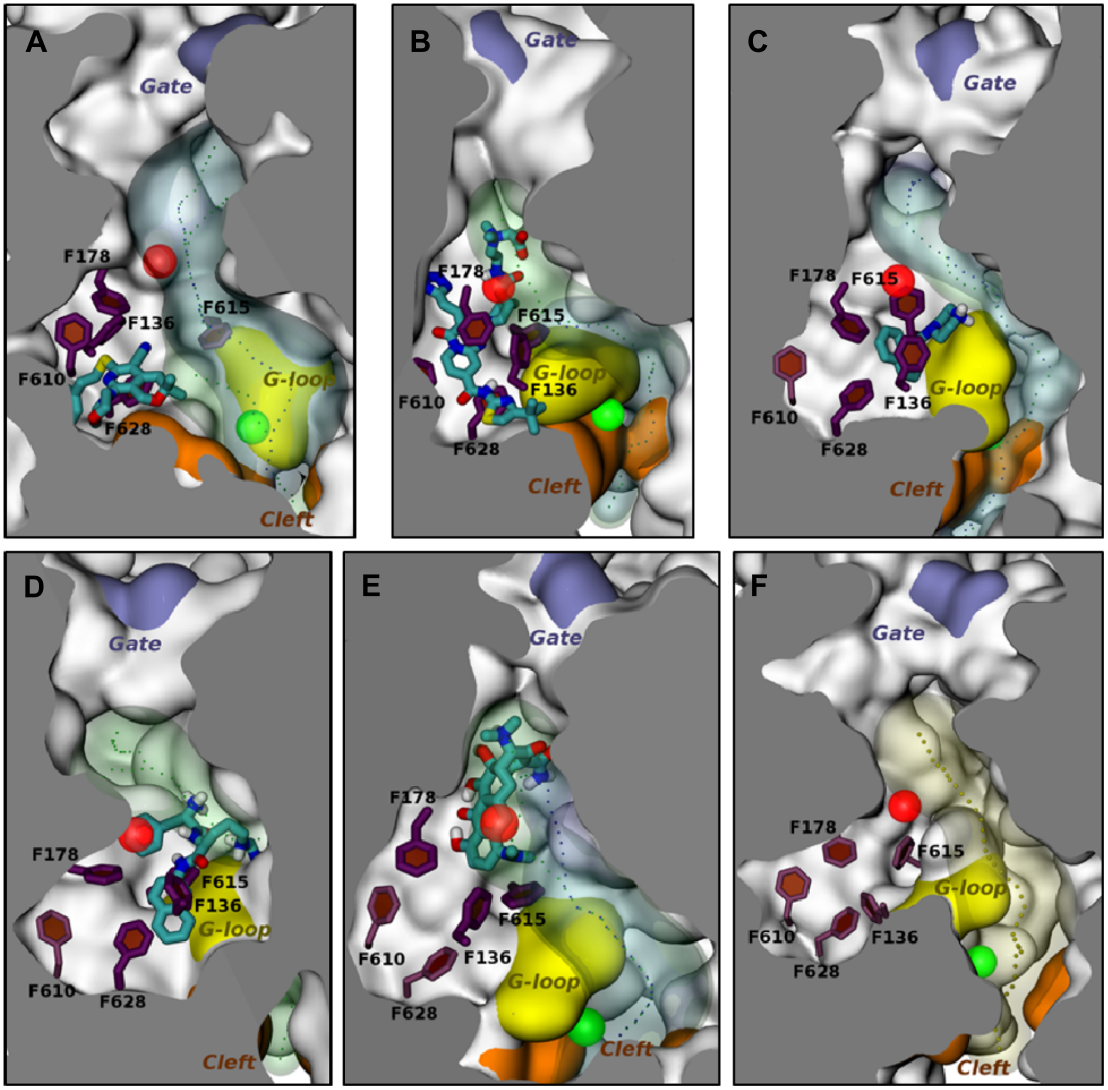
**Molecular dynamic simulations of efflux pump inhibitors and minocycline (MIN) to the distal substrate binding pocket of the Binding protomer of AcrB**. The position of the various ligands used in this study with respect to the hydrophobic trap in protomer B in representative average structures of the complexes from MD simulations are shown. The structures of the ligands are depicted as stick drawings that colored according to the atom type (red, oxygen; yellow, sulfur; dark blue, nitrogen; cyan, carbon; white, hydrogen). The side chains of residues constituting the hydrophobic trap are depicted as stick drawings (thick if the residue is within 3.5 Å of the ligand, thinner otherwise). The rest of the protein is shown with molecular surface, colored in orange, yellow and iceblue at the PC1/PC2 Cleft, the G-loop tip and the exit Gate, respectively, and white elsewhere. The channels leading to the proximity of the exit Gate (see **Figure 1**) and passing through residues of the DP are also shown in the presence and in the absence of the ligand, respectively, are shown as blue and green transparent surfaces. The centers of gravity of the points defining them are shown with points. The centers of mass of the access binding pocket (AP) and of the distal pocket (DP) are shown with green and red transparent spheres, respectively. No contiguous substrate translocation channel was found in the AcrB-PAβN complex **(D)**. The ligands are shown in the following panels: **(A)** MBX2319; **(B)** D13-9001; **(C)** NMP; **(D)** PAβN; **(E)** MIN. **(F)** The substrate translocation channel of AcrB is shown in the ligand-free state to illustrate the representative average structure of the transporter. This figure was taken from Vargiu et al. (2014) and was modified slightly.

Computational models have also been used to study the interactions between EPIs and AcrB. The docking studies reported by Takatsuka et al. (2010) included the EPI NMP, which was predicted to bind to the “cave” region (see above) of distal binding pocket. Because of the low resolution of the docking model, Vargiu and Nikaido (2012) used MD simulations to study the interaction of NMP and PAβN with AcrB (see **Figures 3C,D**). The results of the MD simulations for the EPIs NMP and PAβN were markedly different from those of the substrate compounds that were included in the study. The EPIs were predicted to bind to a site that included the so-called “G-loop” or “switch loop” (Nakashima et al., 2011; Eicher et al., 2012; Cha et al., 2014), which separates the distal and the proximal binding sites and is thought to be involved in the movement of substrates from the proximal to the distal site. In addition, the high resolution of the MD simulations enabled the identification of the amino acid residues that contributed to the binding energy. Recently, MD simulations have been used to evaluate the interaction between MBX2319 (see **Figure 3A**), a novel pyranopyridine EPI, and AcrB (Opperman et al., 2014). MBX2319 is predicted to bind to the same “hydrophobic trap” that was identified in the X-ray cocrystal structure of D13-9001 and AcrB (Nakashima et al., 2013). The data suggests that these compounds may share a common MOA. Based on the ΔG_b_ values, the relative binding affinities of the EPIs were as follows: D13-9001 > MBX2319 > NMP and PAβN, which is consistent with their relative potencies as EPIs. Finally, the computational protocol was able to reproduce the binding of minocycline and D13-9001 to AcrB that was observed in co-crystal structures with RMSD values of 2.7 ± 1.0 and 2.8 ± 0.4 Å (see **Figures 3B,E**), respectively, which increases the level confidence in the modeling results. Therefore, MD simulation is a promising approach to generate molecular hypotheses for the MOA of EPIs.

### An Overview of the Major Classes of EPIs and their Mechanism of Action

In this section, we will briefly review the important classes of EPIs that are active against the RND-family efflux pumps of Gram-negative bacteria. This section provides an overview of the biological activities of each EPI class and drug development-related issues (where appropriate). In addition, we will review the mechanistic action studies for each compound class.

#### Peptidomimetic EPIs: PAβN and the *C*-Capped Dipeptide Analogs

**FIGURE 4:**
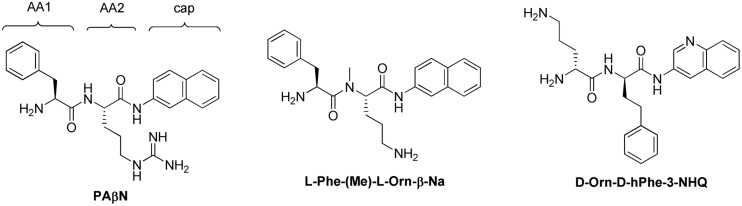
**Structures of PAβN and selected analogs**.

Renau et al. (1999), researchers at Microcide Pharmaceuticals and Daiichi Pharmaceutical Co. reported their discovery of the first inhibitor of RND transporters of Gram-negative bacteria, phenylalanyl arginyl β-naphthylamide (MC-207,110 or PAβN, see **Figure 4**). This dipeptide compound was identified as a promising hit from a screening of 200,000 samples for small molecules that potentiate the antibacterial activity of levofloxacin against strains of *P. aeruginosa* overexpressing MexAB, MexCD, and MexEF pumps.

##### *In vitro*/spectrum of co-antibiotics

The EPI activity of PAβN is strongly dependent on the nature of the antibiotic used as an indicator of pump activity, as measured as a shift in the MIC of the antibiotic (Lomovskaya et al., 2001). Specifically, at 20 μM concentration, the potentiating effect of PAβN on levofloxacin and erythromycin against a strain overexpressing MexAB-OprM was comparable to that of a MexAB-OprM deficient strain. It also exhibited strong potentiation for chloramphenicol, but only weak potentiation for carbenicillin, and none for ethidium bromide, one of the known substrates of MexAB-OprM pump. PAβN did not potentiate antibiotics that are not substrates of MexAB-OprM pump, such as gentamicin and imipenem.

Other biological assays (Lomovskaya et al., 2001) showed that PAβN also inhibits efflux of the AcrAB-TolC pump of *E. coli*, however, it is not very potent against this organism (Opperman et al., 2014). While PAβN did not affect the proton gradient across the inner membrane, it increased permeability of the outer membrane of the PAM2035 strain, which lacks the functional MexAB-OprM pump, in a dose dependent manner. The permeabilizing effect of PAβN was due to its dicationic character. However, the outer membrane activity was abolished by addition of magnesium salt. Although the effect on membrane integrity of PAβN is less than that of polymyxin B, it is a toxicity concern.

##### Scaffold optimization

Because PAβN is not very potent and unstable in murine and human serum, Renau et al. (1999, 2001) prepared over 500 analogs of PAβN aiming to improve the potency (measured by Minimum Potentiation Concentration that decreases the MIC by 8-fold (MPC8) with levofloxacin) and serum stability. For structural optimization, the scaffold was divided into three parts: amino acid 1 and 2 (AA1, AA2) and the (amide) cap (**Figure 4**). Some of the main findings are: (i) an amino acid containing a basic side chain (as either AA1 or AA2) is needed for activity; (ii) Orn could replace Arg; (iii) replacement of Phe with homoPhe led to improved potency; (iv) methylation of the NH that links AA1 and AA2 led to increased serum stability; (v) replacement of L-amino acid with D-amino acid is acceptable and led to increased serum stability; (vi) replacing β-aminonaphthalene with 3-aminoquinoline as the cap group led to slightly reduced potency, but also less intrinsic antibacterial activity and less cytotoxic to mammalian cells. Overall, the analogs did not seem to be much more potent than PAβN (the lowest MPC8 = 2.5 μM for some compounds), but the stability in serum was significantly improved, enabling *in vivo* testing.

##### *In vivo* activity

Two analogs were selected for *in vivo* efficacy testing (**Figure 4**) that showed significant reduction on the growth of *P. aeruginosa* in a murine neutropenic thigh model when used in combination with levofloxacin (Renau et al., 1999, 2001). Specifically, compound L-Phe-(Me)-L-Orn-β-Na at 30 mg/kg (intraperitoneally) in combination with levofloxacin (30 mg/kg, subcutaneously) led to 3-log reduction in colony forming units (cfu) for approximately 4 h, followed by regrowth. A single dose of either levofloxacin or test compound resulted in growth similar to the untreated controls. The immune-suppressed mice in this experiment were infected with *P. aeruginosa* PAM 1032 (1.0 × 10^5^ cfu, intramuscularly).

##### Mechanism of action

The vast majority of the MOA studies for this class of EPIs has been done using PAβN. Lomovskaya et al. (2001) demonstrated that PAβN is a substrate for RND pumps of *P. aeruginosa* (MexB, MexD, and MexF), suggesting that it acts as a competitive inhibitor of substrate binding and/or extrusion. PAβN has been shown to increase the susceptibility of a MexAB-OprM-overproducing strain of *P. aeruginosa* to a wide range of antibiotics, such as levofloxacin, sparfloxacin, chloramphenicol, and erythromycin. However, it was significantly less effective when combined with tetracycline, carbenicillin, and ethidum bromide. If PAβN is a competitive inhibitor, the spectrum of antibiotic potentiation may suggest that the binding sites of PAβN may not overlap with these compounds. To date, there have been no reports of the isolation of mutants with reduced susceptibility to PAβN with mutations that map to an RND-encoding pump gene.

Because of the lack of experimental evidence to shed light on the molecular mechanism of the EPI activity of PAβN, Vargiu and Nikaido (2012) used MD simulations to examine the interaction between PAβN and AcrB. As mentioned above, the results of MD simulation experiments predict that PAβN binds with a relatively low affinity to the distal substrate binding site of AcrB and straddles the G-loop. PAβN is predicted to interact mainly with the hydrophobic residues F136, F178, F615, and F628, but may interact with the hydrophilic residues Q176 and E673. Binding of PAβN is predicted to cause a conformational change that shrinks the substrate extrusion channel in the distal binding pocket, which is where many substrates bind. Therefore, the results suggest two possible mechanisms, which may not be mutually exclusive. First, PAβN prevents movement of the G-loop, which plays an important role in the movement of substrates from the proximal to the distal binding sites (Eicher et al., 2012; Cha et al., 2014). Second, the conformational change in the substrate extrusion channel prevents the binding of other substrates to this site. A docking study suggested that the conformational change induced by PAβN reduced the affinity of minocycline (Vargiu et al., 2014), which supports the second possible MOA.

The first detailed description of the biological activity of PAβN demonstrated that it has the additional MOA of altering the permeability of the outer membrane (Lomovskaya et al., 2001), although PAβN was less potent (half maximal effect at ~70 μg/ml) than the polymyxin B nonapeptide (PMBN), an outer membrane-specific permeabilizing agent (Vaara and Vaara, 1983) that was used as a positive control. The increase in outer membrane permeability is expected to result in increased influx of antibiotics into the periplasm, which would increase susceptibility to an antibiotic substrate in an efflux-independent manner. However, the outer membrane activity of PAβN was abolished by the addition of 1 mM Mg^2+^, but it is not clear whether Mg^2+^ was effective at higher concentrations of PAβN. The outer membrane activity of PAβN and the effect Mg^2+^ was also described recently by Lamers et al. (2013). Therefore, experiments with PAβN should include 1 mM Mg^2+^ to minimize the effect of the outer membrane activity.

#### 1-(1-Naphthylmethyl)-Piperazine (NMP) and the Arylpiperazine Analogs

**FIGURE 5:**
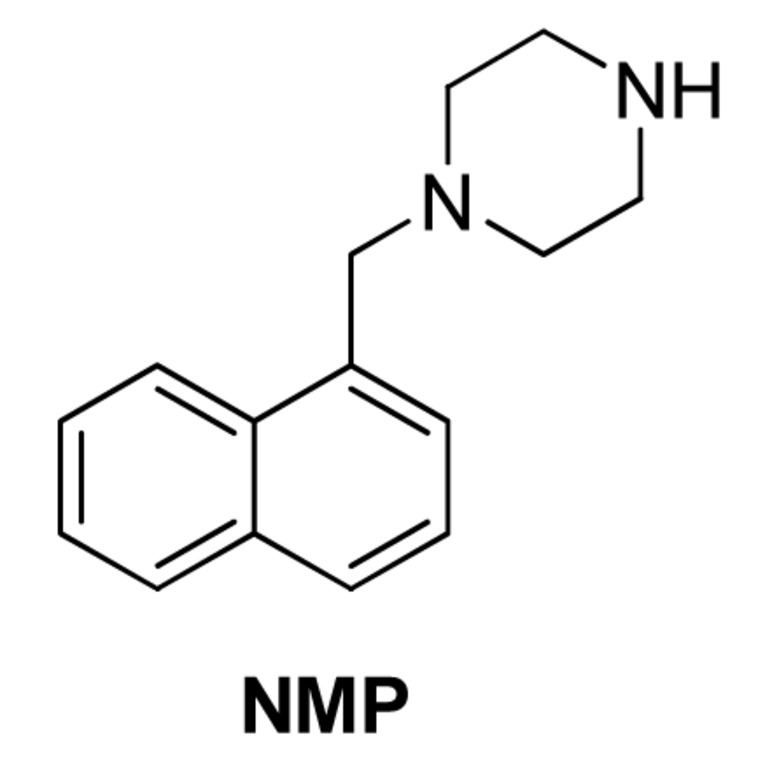
**Structure of NMP**.

Bohnert and Kern (2005) reported the discovery of 1-(1-Naphthylmethyl)-piperazine (NMP, **Figure 5**) as an EPI against *E. coli*. The authors screened a library of *N*-heterocyclic compound library for potentiators of levofloxacin against *E. coli* strains overexpressing *acrAB* and *acrEF* (2-DC14PS and 3-AG100MKX, respectively) and found some phenylpiperazine derivatives with activity. Further SAR study led to the synthesis of NMP, one of the most potent analogs. NMP (at 100 μg/mL) caused an increase in the intracellular accumulation of levofloxacin. It also caused accumulation of ethidium bromide in a dose dependent manner (6.25–100 μg/mL) in the pump overexpressing strains (2-DC14PS and HS414), but not in the pump-deficient strains (1-DC14PS and HS276), suggesting it was an inhibitor of the AcrAB and AcrEF efflux pumps. Because of low potency and the possibility that NMP acts as serotonin agonist, the likelihood that this EPI will be developed further as a adjunctive therapeutic agent is low (Zechini and Versace, 2009).

At 100 μg/mL concentration, against *E. coli* strains overexpressing *acrAB* or *acrEF*, NMP potentiated levofloxacin by 8- to 16-fold, and by 4- to 8-fold for oxacillin, rifampin, chloramphenicol, and clarithromycin. It reduced the MIC of linezolid against the *acrEF*-overexpressing strain (2-DC14PS) by 32-fold, and by 8-fold against the *acrAB*-overexpressing strain (3-AG100MKX). Other fluoroquinolones (ciprofloxacin, norfloxacin, enoxacin, and pefloxacin), erythromycin, azithromycin, clindamycin, doxycycline, and nitrofurantoin, but not the ketolide telithromycin, glycopeptides, aminoglycosides, trimethoprim-sulfamethoxazole, and fosfomycin were potentiated by NMP (at 100 μg/mL), as indicated by ≥4-fold reduction of MIC against the 3-AG100MKX strain.

Testing against 60 clinical isolates of *E. coli* using NMP at 100 μg/mL showed that this compound was moderately active in reversing MDR in clinical isolates of *E. coli* and could partially restore fluoroquinolone susceptibility (Kern et al., 2006). NMP caused reduction of MICs of levofloxacin, linezolid and ethidium bromide by ≥4-fold in >50% of the isolates. Although its potentiation of linezolid was notable, the effect was not enough to make the isolates susceptible to this antibiotic. NMP also showed partial MDR reversal effects against other *Enterobacteriaceae* species, e.g., *E. aerogenes* and *K. pneumonia* (Schumacher et al., 2006), and *Acinetobacter baumannii* (Pannek et al., 2006). It was noticed that NMP and PAβN showed markedly different preferences for antibacterial agents, as well as, the isolates that each could work with, suggesting they might inhibit different pumps, bind to different sites of the same pump(s), or act on different target(s).

##### Mechanism of action

1-(1-Naphthylmethyl)-piperazine is widely used as a research reagent. Consequently, there is great interest in the MOA of this compound. Schuster et al. (2014) were the first group to successfully isolate AcrB mutants resistant to NMP. They screened a library of mutants in which the substrate binding and extrusion domain of AcrB were randomly mutagenized using error-prone PCR for mutants that were able to form colonies on plates containing NMP and linezolid. The authors isolated several mutants with reduced susceptibility to NMP that did not decrease function of the pump. Site-directed mutagenesis of wild-type *acrB* was used to verify the genetic basis for the NMP-resistant phenotype. Only a double mutant, in which Gly141 was substituted with Asp (G141D) and Asn282 was substituted with Tyr (N282Y), significantly affected susceptibility to NMP. The mutated residues are located near the outer face of the distal substrate binding pocket near Phe610 (see **Figure 1A**), which plays an key role in the extrusion process (Bohnert et al., 2008) and is across from the G-loop that separates the proximal (access) and the distal (substrate) binding sites (Nakashima et al., 2011; Eicher et al., 2012). The authors speculate that G141D N282Y amino acid substitutions prevent binding of NMP at a site near Phe610, where NMP is predicted to alter the specificity or the conformational changes of the binding pocket by acting on Phe610 in a non-competitive manner (see below). Interestingly, the NMP resistant phenotype was observed only in the presence of linezolid, and partial resistance in the presence of Hoechst 33342 and levofloxacin. The reason for this is unclear. However, as mentioned above, it is possible that the NMP resistant mutant enables the extrusion of linezolid when NMP is bound to a site that straddles the G-loop, which was predicted by MD simulations (see **Figure 3C**; Vargiu and Nikaido, 2012; Vargiu et al., 2014). The estimated binding energy is -10.6 ± 7.9 kcal/mol. The NMP binding site includes interactions with hydrophobic residues near the hydrophobic patch (F664 and F666) and G617 of the G-loop. This suggests the NMP inhibits the action of AcrB by interfering with the movement of the G-loop, which has been shown to play important role in the extrusion of certain substrates (Eicher et al., 2012; Cha et al., 2014). As shown in **Figure 1A**, the binding site for NMP that was predicted by MD simulations does not include G141 and N282. Also, as is the case for PAβN, NMP is predicted to induce a conformational change that results in a reduction of the width of the substrate binding cleft (Vargiu and Nikaido, 2012). Therefore, the amino acid substitutions in the NMP resistant mutant may prevent these NMP-induced conformational changes, allowing extrusion of linezolid when NMP is bound.

Despite the fact that NMP is used widely as a research reagent, two important questions about its activity have not been resolved. It has been stated in the literature that NMP is not a substrate of RND-family pumps, however, convincing data to support this statement have not been published. For example, Schuster et al. (2014) reported that the rate of intracellular accumulation of NMP (100 μg/ml) was similar in an AcrB overexpressing strain, an AcrB-deficient strain, and NMP resistant mutants. Based on this result, the authors concluded that NMP is not a substrate (or a poor substrate) of AcrB (Schuster et al., 2014). However, the data was not presented in their paper. As discussed in the next section, these types of assays must be interpreted with caution when high concentrations of an EPI are present in the medium. Therefore, is not a trivial matter to determine whether an EPI is a substrate or non-substrate of an RND-family pump. Unlike PAβN, additional mechanisms of action for NMP have not been reported. However, NMP exhibits antibacterial activity at a concentration of 400 μg/ml (Bohnert and Kern, 2005), which is only 4-fold higher than the concentration at which it is used as an EPI. This indicates that this compound acts on an additional cellular target, resulting in growth inhibition. The most likely secondary target of NMP is the membrane, however, the effect this compound on membrane potential or integrity has not been reported.

#### D13-9001 and the Pyridopyrimidinone Analogs

**FIGURE 6:**
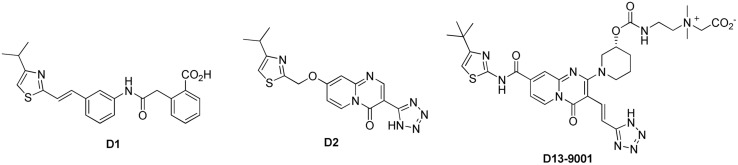
**Structure of D13-9001 and its developmental precursors**.

Yoshida et al. (2007) at Daiichi Pharmaceutical Co. and Essential Therapeutics described D13-9001 (**Figure 6**) as a MexAB-OprM specific pump inhibitor against *P. aeruginosa* with good solubility and *in vivo* activity. This report was the last of a seven article series starting from 2003, describing a systematic optimization of another screening hit compound (**D1**, **Figure 6**; Nakayama et al., 2003a,b, 2004a,b; Yoshida et al., 2006a,b). Compound **D1** only inhibited MexAB-OprM pump, and not the MexCD-OprJ, MexEF-OprN, or MexXY-OprM pumps. It exhibited very good MPC8 with levofloxacin (≤0.63 μM) against a *P. aeruginosa* strain (PAM1723) that has overexpressed MexAB-OprM and disrupted MexCD-OprJ and MexEF-OprN. However, this compound was practically insoluble in water and showed no efficacy *in vivo*.

The poor *in vivo* performance of **D1** was attributed to its high affinity for serum albumin (>98% protein bound) and the increase in MPC8 (>10-fold shift) when serum was added. After some unsuccessful attempts at conservatively modifying the scaffold to improve pharmacokinetic properties, big changes were made by installing the quinolone and pyridopyrimidinone fragments to the mid-section of the scaffold. The pyridopyrimidinone analogs, such as **D2**, displayed higher MPC8 as compared to **D1** (5 μg/mL for aztreonam and 10 μg/mL for levofloxacin), but had lower affinity to serum protein and were effective *in vivo* (Nakayama et al., 2003b). Subsequent efforts to further optimize the scaffold for activity and pharmacokinetic properties led to the synthesis of D13-9001. This zwitterionic compound was soluble enough for *iv* administration (747 μg/mL in pH 6.8 buffer), exhibited MPC8 = 2 μg/mL for levofloxacin and aztreonam against the PAM1723 strain of *P. aeruginosa*. D13-9001 demonstrated acceptable PK profiles in rats and monkeys. It did not alter the serum level of aztreonam when both were given by *iv* (1000 mg/kg of aztreonam T, 1.25–20 mg/kg of D13-9001, 2 h infusion). In a lethal pneumonia model in rats (using PAM1020 strain), in combination with aztreonam (1000 mg/kg) D13-9001 (at 1.25–20 mg/kg) showed improved survival rates at the end of day seven.

##### Mechanism of action

Nakashima et al. (2013) reported that D13-9001 does not perturb the inner or the outer membranes. In this same paper, the authors determined that D13-9001 is not a substrate of AcrB. They measured the time-dependent change in concentration of D13-9001 in the media containing either an AcrB overexpressing or an AcrB-deficient strain. Compounds that are not substrates accumulate in both strains, resulting in a decrease compound concentration in the media, while pump substrates will accumulate only in the AcrB-deficient strain. Using this assay, the authors concluded that D13-9001 is not a substrate. However, results of this nature should be interpreted with caution when the test compound is a potent EPI and is present at a high concentration (28.6 μM) in the medium. The high concentration of EPI is likely to inhibit AcrB, causing compound to accumulate in the AcrB overexpressing strain. Thus, potent EPIs would appear to be non-substrates, even when they are actually pump substrates. At this point, it is not clear whether D13-9001 is a non-substrate. This situation underscores the difficulties at present in determining whether EPIs are substrates using the available assays.

As mentioned above, Nakashima et al. (2013) elucidated the three-dimensional structure of the EPI D13-9001 bound to AcrB and MexB. The hydrophobic tert-butyl thiazolyl aminocarboxyl pyridopyrimidine moiety of D13-9001 binds tightly to a narrow depression, referred to as the hydrophobic trap, in the deep substrate binding pocket in the “Binding” protomer (see **Figures 2A,B**). The hydrophobic trap branches off from the substrate translocation channel and is lined with hydrophobic residues (F136, F178, F610, F615, and F628). In addition, the tetrazole ring and the piperidine aceto-amino ethylene ammonio-acetate moiety interact with ionic and/or hydrophilic residues (N274, R620, Q176, and S180 in addition to aliphatic residues (I277 and L177) that are in the substrate translocation channel. The piperidine aceto-amino ethylene ammonio-acetate moiety extends into the substrate binding sites of both minocycline and doxorubicin. The crystal structure of the D13-9001 binding site in MexB was found to be similar to the binding site in AcrB, except that the conformation of the PAEA moiety in the substrate translocation channel was different. A homology model of the three-dimensional structure of MexY predicted that D13-9001 would not bind because F178 at the edge of the hydrophobic trap of AcrB/MexB has been replaced with a bulky W177 in MexY, which is predicted to prevent binding by steric hindrance. The model was confirmed when a site-directed mutant of MexY (W177F) was shown to be sensitive to inhibition by D13-9001. Conversely, D13-9001 does not bind to AcrB (F178W). As shown by ITC, D13-9001 binds tightly to AcrB and MexB with K_D_ values of 1.15 and 3.57 μM, respectively. It is possible that the successful production of co-crystals with D13-9001 is due to the high affinity of this compound for AcrB and MexB. This EPI inhibits efflux of a broad range of compounds, presumably not by competitive inhibition (yet to be determined experimentally). The current hypothesis is that D13-9001 binds tightly to the hydrophobic trap and prevents the conformational changes that are needed for the proper activity of the pump (Nakashima et al., 2013). In addition, the presence of the hydrophilic portion of this EPI in the substrate binding channel is predicted to prevent substrate binding to this site. Therefore, this study has made a significant advance in the understanding of the mechanism of inhibition of an EPI, and has identified a previously unknown binding site in RND pumps that can be exploited for the rational design or optimization of EPIs. Because MD simulations (see **Figure 3B**) have been able to reproduce the structure of D13-9001 bound to AcrB (rmsd 2.8 Å; Vargiu et al., 2014), this tool may prove to be useful in evaluating novel EPIs.

#### Pyranopyridines (MBX2319)

**FIGURE 7:**
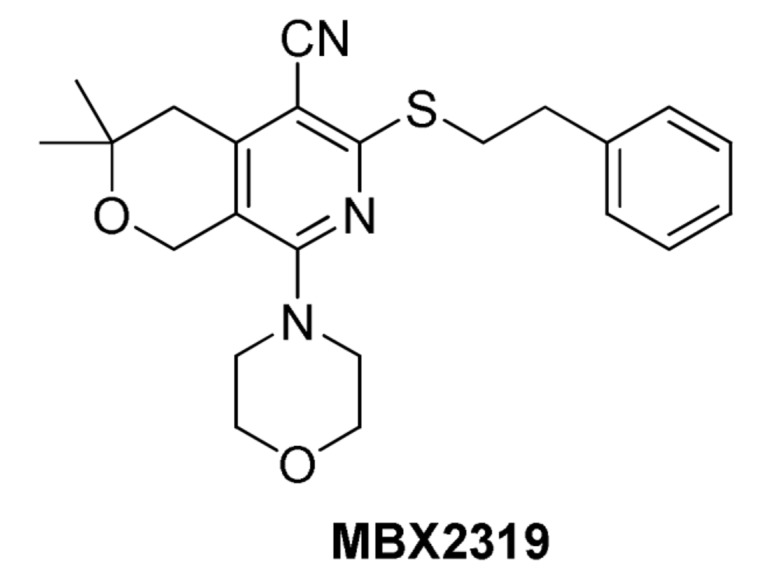
**Structure of MBX2319**.

Opperman et al. (2014) at Microbiotix reported compound MBX2319 (**Figure 7**) as a novel inhibitor of the AcrAB efflux pump of *E. coli*. It was discovered from a high-throughput screening campaign designed to look for small molecules that potentiate ciprofloxacin against *E. coli*. Because the authors were not specifically looking for efflux inhibitors, the bacterial strain was not engineered to overexpress any transporters. MBX2319 had no intrinsic antibacterial activity (MIC ≥ 100 μg/mL), but exerted significant potentiating effects on antibiotics that are substrates of AcrB, such as fluoroquinolones, β-lactams, chloramphenicol, erythromycin, and linezolid at concentrations of 3.1–12.5 μg/ml. Consistent with the proposed mechanism, MBX2319 did not affect MICs of antibiotics against *acrB-*deleted mutant or of antibiotics that are not AcrB pump substrates, e.g., gentamicin and carbenicillin. Further, MBX2319 caused increased intracellular accumulation of dye Hoechst 33342 and nitrocefin in a dose dependent manner in wild-type *E. coli* strains, but not the Δ*acrB* and Δ*tolC* strains. This inhibitor also worked against other *Enterobacteriaceae* species, i.e., *Shigella flexneri*, *K. pneumoniae*, *S. enterica* and *E. cloacae*. Against *P. aeruginosa* (ATCC 27854), MBX2319 reduced the MIC of cefotaxime (but not ciprofloxacin or levofloxacin) ca. 6-fold. However, the observation that PMBN increases the EPI activity of MBX2319 against *P. aeruginosa* indicates the outer membrane of this organism is the major obstacle to activity (Opperman et al. manuscript in preparation). Therefore, the pyranopyridines have the potential to be used as an adjunctive therapy against both *Enterobacteriaceae* and *P. aeruginosa.*

##### Mechanism of action

Based on the results of MD simulations, MBX2319 is predicted to bind to the same “hydrophobic trap” (see **Figure 3A**) that was identified in the X-ray crystal structure of D13-9001 bound to AcrB (Nakashima et al., 2013). The pyridine ring of MBX2319 is predicted to make a ring-stacking interaction with the rings of F628 on one side and F136 on the other. The morpholinyl moiety is predicted to be in close proximity to Y327, and the phenylethylthiol group is loosely located in a hydrophobic pocket surrounded by F610, F628, P326, and Y327 (see **Figure 3A**). The prevalence of interactions with aromatic or aliphatic residues results in a calculated ΔG_b_ of -12.5 kcal/mol. Indeed, almost 70% of ΔG_solv_ comes from residues belonging to the hydrophobic trap. As this region undergoes large conformational changes during the functional rotation of AcrB, binding of MBX2319 with high affinity could hinder these rearrangements (or those triggering the translocation of protons from the periplasm to the cytoplasm upon substrate binding). In addition, as a result of the interaction with the hydrophobic trap, the part of the distal binding site that forms a cleft, which is the binding site for minocycline and doxorubicin appears to shrink (compare **Figures 3A–F**), which may inhibit substrate binding. Indeed, results from the cell-based nitrocefin efflux assay (described above) demonstrated that at 0.2 μM MBX2319 increased the apparent Km of AcrB for nitrocefin by >4-fold (Opperman et al., 2014), suggesting, that the EPI altered the affinity of the substrate for AcrB. Thus, the results of MD simulations are consistent with those of crystallographic methods and can be useful for the generation of molecular hypotheses for the MOA of EPIs that can be tested using genetic and biochemical experiments.

The EPI activity of MBX2319 appears to be specific for RND pump inhibition. MBX2319 does not exhibit intrinsic antibacterial activity (MIC ≥ 100 μM), so it is unlikely to act on a secondary non-specific target, such as the inner or outer membrane. This possibility is supported by experimental results that demonstrate MBX2319 does not perturb the proton gradient or integrity of the inner membrane, nor does it increase the permeability of the OM (Opperman et al., 2014).

## Future

Recent advances in methodologies have led to a greater understanding of the MOA of EPIs. In particular, the elucidation of a three-dimensional structure of the EPI D13-9001 bound to AcrB has resulted in hypotheses for the MOA of this compound. In addition, this structure has identified a previously unknown binding site of EPIs in the distal binding pocket, known as the hydrophobic pocket, which can be exploited for the rational design and optimization of novel EPIs with improved potency and spectrum of activity. Due to recent advances in technology, MD simulations have been able to reproduce the binding of two substrates and an EPI elucidated by crystallographic studies with a high degree of accuracy. This raises the possibility that this method could be used to evaluate novel inhibitors. Together, these advances could be useful in overcoming some of the major challenges in the discovery and development of clinically useful EPIs. One of the major challenges in the design of EPIs for Gram-negative is to make compounds that are able to traverse the OM and can bind to the RND pumps with high affinity. Penetration of the OM is controlled by porins, which prefer smaller hydrophilic and zwitterionic molecules. In contrast, the major RND pumps prefer larger hydrophobic molecules. By using rational drug design, however, it will be possible to identify suitable positions on EPIs to append charged groups that will improve OM penetration but will not decrease affinity for the pump.

## Conflict of Interest Statement

Timothy J. Opperman and Son T. Nguyen are employees of Microbiotix, Inc.
